# Derivation and validation of a nutrition-covered prognostic scoring system for extranodal NK/T-cell lymphoma

**DOI:** 10.3389/fnut.2023.1080181

**Published:** 2023-05-12

**Authors:** Tiange Lu, Xue Shi, Xueling Ge, Ying Li, Yiqing Cai, Xiaomin Chen, Shunfeng Hu, Mei Ding, Xiaosheng Fang, Fang Liu, Xiangxiang Zhou, Xin Wang

**Affiliations:** ^1^Department of Hematology, Shandong Provincial Hospital, Cheeloo College of Medicine, Shandong University, Jinan, Shandong, China; ^2^Department of Hematology, Shandong Provincial Hospital Affiliated to Shandong First Medical University, Jinan, Shandong, China; ^3^School of Medicine, Shandong University, Jinan, Shandong, China; ^4^Department of Hematology, The Affiliated Hospital of Qingdao University, Qingdao, Shandong, China; ^5^Shandong Provincial Engineering Research Center of Lymphoma, Jinan, Shandong, China; ^6^Branch of National Clinical Research Center for Hematologic Diseases, Jinan, Shandong, China; ^7^National Clinical Research Center for Hematologic Diseases, The First Affiliated Hospital of Soochow University, Suzhou, China; ^8^Department of Psychiatry, Campbell Family Mental Health Research Institute, Centre for Addiction and Mental Health, University of Toronto, Toronto, ON, Canada

**Keywords:** nutrition, CONUT score, extranodal NK/T-cell lymphoma (ENKTCL), prognosis, model

## Abstract

**Introduction:**

Patients with aggressive lymphomas are at high risk of losing body resources, resulting in malnutrition, immunodeficiency and inferior outcomes. Nutritional status is closely associated with survival, but often neglected in the prognostic assessment. This study aimed to explore the significance of nutritional status in extranodal NK/T-cell lymphoma (ENKTL).

**Methods:**

Univariate and multivariate Cox regression analyses were conducted to examine the significance of nutritional index on overall survival (OS) and progression-free survival (PFS). A nutrition-incorporated score system was constructed based on the multivariate results, and its calibration, discrimination and clinical utility were tested in the training and validation cohort.

**Results:**

Multivariate analysis revealed controlling nutritional status (CONUT) score could independently predict OS (HR 10.247, *P*=0.001) and PFS (HR 5.587, *P*=0.001) in addition to prognostic index of natural killer lymphoma plus EBV (PINK-E). Herein, a reformative model, CONUT-PINK-E, was developed and further verified in external validation cohort. CONUT-PINK-E classified patients into three risk grades with significant survival differences (*P* < 0.001). Compared with the current models, CONUT-PINK-E presented superior discrimination, calibration and clinical benefit.

**Discussion:**

In this study, we firstly verified that CONUT score was efficient to screen prognosis-related malnutrition in ENKTL. Moreover, we developed the first nutritional assessment-covered scoring system, CONUT-PINK-E, which might be a promising tool to provide references for clinical decision-making of ENKTL patients.

## Introduction

Extranodal natural killer (NK)/T-cell lymphoma, nasal type (ENKTL), derived from NK cells or cytotoxic T cells, is a unique and uncommon clinicopathological entity of non-Hodgkin lymphoma (NHL), more prevalent in East Asia and Latin America (1, 2). ENKTL is distinguished by Epstein–Barr virus (EBV) infection and upper aerodigestive tract (UADT) involvement, and is highly aggressive with poor survival outcomes (1, 3, 4).

Since body resources are consuming, malnutrition is a common phenomenon in patients with advanced malignancies, accompanied with compromised immune competence, degressive physical activity and worsened clinical outcomes (5). Even though the prevalence and severity in ENKTL remain unclear, ENKTL patients are at high malnutrition risk due to the primary location and tumor burden (6, 7). Nevertheless, nutritional status assessment has often been neglected in prognosis evaluation.

Nutritional status closely affects the response to antineoplastic therapy, tightly associated with therapy intensity and treatment-related toxicity (8, 9). Either reduced intensity or unbearable toxicity badly impairs the treatment benefits and shortens patients’ survival (10). Since cure remains less promising for most ENKTL patients in advanced stage, optimal supportive care is essential to tolerate long-term treatments and achieve a prolonged survival (5). The supportive care relies on the accurate nutritional status assessment, so it is urgent to explore the applicable nutritional index for ENKTL patients.

In last few decades, nutrition-related indices constantly spring up and nutritional status evaluation attracts increasing attention in multiple cancers, such as head and neck cancers and gastric cancer (11, 12). Prognostic nutritional index (PNI) and controlling nutritional status (CONUT) score, two emerging nutritional indices, have been verified possessing greater prognostic significance (13, 14) than the traditional nutritional parameters, such as body weight, triceps skin fold thickness, mid-arm muscle circumference and body mass index (BMI) (15, 16). PNI is calculated as serum albumin level (ALB, g/L) + 0.005 × absolute lymphocyte count (ALC, per mm^3^) (17) and CONUT score is calculated from the serum ALB, ALC and total cholesterol (TC) (12), both reflecting the long-term nutritional and immune response status (12, 18).

Nevertheless, the association between PNI, CONUT score and the survival outcomes of ENKTL remains undiscovered. This study aimed to examine the potential of PNI and CONUT score serving as a prognostic marker in ENKTL and further establish a nutrition evaluation-incorporated risk stratification system. The performance of the reformative model would be verified from multiple dimensions and tested in an external validation cohort.

## Patients and methods

### Study population

We retrospectively analyzed newly diagnosed ENKTL patients in two centers of China, patients hospitalized at Shandong Provincial Hospital from January 2011 to June 2020 constituting the training cohort and patients treated at the Affiliated Hospital of Qingdao University from January 2013 to June 2020 forming the external validation cohort. Two cohorts followed the same inclusion and exclusion criteria. The inclusion criteria were extranodal NK/T-cell lymphoma diagnosed by biopsy based on the WHO 2016 Classifications of mature lymphoid, histiocytic and dendritic neoplasms (19) and treatment-naïve. The key exclusion criteria were with incomplete clinical data and follow-up information or a history of other malignancies or major disease.

### Data collection

The baseline data, such as gender, age, extranodal sites, bone marrow involvement, local lymph node involvement, distant lymph node involvement, primary site, primary tumor invasion, Eastern Cooperative Oncology Group (ECOG) score, Ann Arbor stage, B symptoms, international prognostic index (IPI), Korean Prognostic Index (KPI), Prognostic index of natural killer lymphoma (PINK) and PINK plus EBV (PINK-E) were gathered. Laboratory examinations, including serum lactate dehydrogenase (LDH), β2-microglobulin (β2-MG), ALB, ALC, TC and EBV DNA copies were collected.

### Criteria of CONUT score

CONUT score criteria were shown in Supplementary Table 1.

### Follow-up

The follow-up data, including therapeutic regimens and survival outcomes, were prospectively collected and retrospectively analyzed. The primary observation endpoint was overall survival (OS), followed by progression-free survival (PFS). OS was defined as the period from the date of diagnosis to the date of last follow-up or all-cause death. PFS was calculated as the interval from diagnosis to the first disease progression or last follow-up.

### Statistical analyses

Continuous variables that did not fit the normal distribution were reported as medians [interquartile range (IQR)] and compared using the Mann–Whitney *U*-test. Normally distributed variables, reported as mean ± (standard deviation), were compared using the Student t test. Categorical data, presented as frequency (%), were compared using the Chi-squared test or Fisher’s exact test. The dichotomous cutoff values of PNI were determined by receiver operating characteristic (ROC) curves according to the maximal associated J statistic (Youden’s index). Cox proportional hazards regression model was used in univariate analyses (UVA) and multivariate analyses (MVA) and the results were presented as hazard ratio (HR) and 95% confidence interval (CI). Variables significantly associated with survival in the univariate analysis (*p* < 0.05) were brought into MVA and the further screened independent variables constituted the novel model, whose point assignment was defined according to the rounded regression coefficients (B). Harrell’s C-statistic was calculated to reflect the predictive discriminability and calibration curves were plotted to estimate the performance of the proposed model. OS and PFS estimated curves were constructed using the Kaplan–Meier method and compared using the log-rank test. Alluvial plot shows the frequency and relationship between the risk grades of the novel model and its included risk factors in the total population of training and validation cohort. Time-dependent ROC curve analysis, decision curve analysis (DCA), net reclassification index (NRI) and integrated discrimination improvement (IDI) were performed to compare the predictive superiority of the novel model and the current scoring systems. *p* < 0.05 was considered statistically significant and all tests were two-tailed. Statistical analyses were executed by SPSS 25.0 (SPSS, Chicago, IL, United States) and R program (version 3.6.2; R Foundation for Statistical Computing, Vienna, Austria). Several packages were used in the R environment, including “rms,” “forestplot,” “CsChange,” “Time-ROC,” “stdca,” “survIDINRI,” “survival,” “survminer,” and “ggalluvial.”

## Results

### Baseline clinical characteristics of cohorts

A total of 160 patients were included in the study, 80 patients in the training cohort and 80 patients in the external validation cohort. The baseline clinical characteristics in two cohorts were presented in Table 1. Most features between the two cohorts were comparable, including onset age (*p* = 0.379), sex distribution (*p* = 0.177), performance status (*p* = 0.205), bone marrow involvement (*p* = 0.786), involved extranodal sites (*p* = 0.088) and UADT involvement (*p* = 0.256). Nevertheless, some differences still existed in some characteristics between the training and validation cohort, such as Ann Arbor stage (III–IV stage, 27.5% vs. 48.8%, *p* = 0.006), LDH level (227.1 vs. 276.2 U/L, *p* = 0.016), IPI grades (*p* = 0.001), KPI grades (*p* = 0.004) and therapy schedules (*p* = 0.012). Patients in advanced stage accounted for a higher proportion in the validation cohort, which might explain the corresponding higher LDH level, more high-risk patients and more patients receiving chemotherapy alone in the validation cohort.

**Table 1:** Basic clinical characteristics of the training and validation cohorts.

Variables	Training cohort	Validation cohort	*P*-value
	(*n* = 80)	(*n* = 80)	
**Basic results**			
Age, years	51.5 (41.5,60.0)	48.0 (37.3,57.8)	0.379
**Sex (%)**			
Female	22 (27.5%)	30 (37.5%)	0.177
Male	58 (72.5%)	50 (62.5%)	
**ECOG score (%)**			
<2	63 (78.8%)	56 (70.0%)	0.205
≥2	17 (21.2%)	24 (30.0%)	
**BM involvement (%)**			
Absence	72 (90.0%)	73 (91.3%)	0.786
Presence	8 (10.0%)	7 (8.7%)	
**Extranodal sites (%)**			
< 2	60 (75%)	50 (62.5%)	0.088
≥2	20 (25%)	30 (37.5%)	
**Ann Arbor stage (%)**			
I/II	58 (72.5%)	41 (51.2%)	0.006
III/IV	22 (27.5%)	39 (48.8%)	
**Primary site (%)**			
UADT	59 (73.8%)	65 (81.3%)	0.256
Non-UADT	21 (26.2%)	15 (18.7%)	
**B symptoms (%)**			
Absence	47 (58.8%)	40 (50.0%)	0.267
Presence	33 (41.2%)	40 (50.0%)	
**Serological results**			
LDH, U/L	227.1 (176.0, 305.5)	276.2 (198.7, 457.6)	0.016
β2-MG, mg/L	2.6 (2.2, 3.6)	2.3 (1.7, 3.1)	0.006
TC, mmol/L	4.4 (3.5, 5.1)	4.1 (3.4, 4.8)	0.079
ALB, g/L	37.3 (32.8, 39.9)	36.2 (32.2, 39.6)	0.354
ALC, 10^9/L	1.4 (0.8, 1.8)	1.3 (0.9, 1.6)	0.582
PNI	45.4 (41.2, 51.0)	45.4 (39.5, 48.2)	0.261
CONUT score	3.0 (1.0, 5.0)	4.0 (2.0, 5.0)	0.164
**Clinical scoring systems**			
IPI (%)			
Low risk (0/1)	46 (57.5%)	26 (32.5%)	0.001
Low-intermediate risk (2)	17 (21.3%)	13 (16.3%)	
Intermediate-high risk (3)	8 (10.0%)	18 (22.5%)	
High risk (4/5)	9 (11.3%)	23 (28.7%)	
**KPI (%)**			
Group1 (0)	13 (16.3%)	16 (20.0%)	0.004
Group2 (1)	29 (36.2%)	18 (22.5%)	
Group3 (2)	21 (26.2%)	10 (12.5%)	
Group4 (3/4)	17 (21.3%)	36 (45.0%)	
**PINK (%)**			
Low risk (0)	24 (30.0%)	12 (15.0%)	0.073
Intermediate risk (1)	25 (31.3%)	32 (40.0%)	
High risk (2/3/4)	31 (38.7%)	36 (45.0%)	
**PINK-E (%)**			
Low risk (0/1)	39 (48.8%)	35 (43.8%)	0.536
Intermediate risk (2)	16 (20.0%)	22 (27.5%)	
High risk (3/4/5)	25 (31.2%)	23 (28.7%)	
**Therapy (%)**			
CT alone	30 (37.5%)	48 (60.0%)	0.012
RT alone	12 (15.0%)	5 (6.3%)	
CRT	38 (47.5%)	27 (33.8%)	

ECOG, Eastern Cooperative Oncology Group; BM, bone marrow; UADT, upper aerodigestive tract; LDH, lactate dehydrogenase; β2-MG, beta-2 microglobulin; TC, serum total cholesterol; ALB, serum albumin; ALC, absolute lymphocyte count; PNI, Prognostic Nutritional Index; CONUT, Controlling Nutritional Status score; IPI, international prognostic index; KPI, Korean Prognostic Index; PINK, Prognostic index of natural killer lymphoma; PINK-E, PINK plus Epstein–Barr virus (EBV); CT, chemotherapy; RT, radiotherapy; CRT, chemoradiotherapy.

### Association between nutritional indices and survival outcomes of ENKTL patients

**Figure 1:**
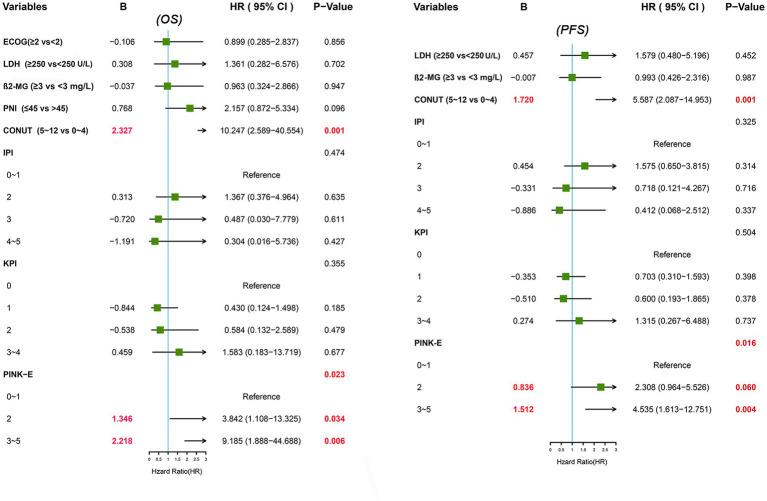
Forest plots of multivariate analysis for OS and PFS.

We further conducted UVA and MVA to investigate the prognostic significance of PNI and CONUT score in ENKTL. UVA illustrated that PNI was an predictive factor to OS (HR [95% CI] = 2.275 [1.184–4.369], *p* = 0.014) but not to PFS (HR [95% CI] =1.653 [0.926–2.954], *p* = 0.089) while CONUT score was a significant predictor to both OS (HR [95% CI] =29.385 [9.583–90.102], *p* < 0.001) and PFS (HR [95% CI] =12.516 [5.899–26.558], *p* < 0.001; Table 2). Then all the significant variables (*p* < 0.05) in UVA were brought into MVA excluding PINK out of the consideration that PINK and PINK-E might have collinearity and PINK-E is a more integrated index. As shown in forest plot (Figure 1), MVA revealed that CONUT score could independently predict OS (HR [95% CI] =10.247 [2.589–40.554], *p* = 0.001) and PFS (HR [95% CI] =5.587 [2.087–14.953], *p* = 0.001) of ENKTL. The CONUT score-estimated Kaplan–Meier survival curves differentiated patients into two groups with distinct OS (*p* < 0.001) and PFS (*p* < 0.001; Supplementary Figure 1), verifying that CONUT score could serve as a competent index to screen prognosis-related malnutrition in ENKTL.

**Table 2:** Univariate analysis of OS and PFS in the training cohort.

Basic results	OS					PFS			
	B	SE	HR (95%CI)	*P*-value		B	SE	HR (95%CI)	*P*-value
Age, ≥60 vs. < 60, years	0.668	0.393	1.951 (0.903–4.217)	0.089		0.411	0.342	1.508 (0.771–2.951)	0.230
Sex, male vs. female	−0.218	0.370	0.804 (0.390–1.659)	0.555		−0.069	0.319	0.933 (−0.500–1.742)	0.828
ECOG score, ≥2 vs. < 2	0.820	0.362	2.271 (1.117–4.620)	**0.024**		0.618	0.322	1.855 (0.986–3.489)	0.055
BM involvement, presence vs. absence	0.876	0.450	2.401 (0.995–5.797)	0.051		0.471	0.437	1.601 (0.680–3.772)	0.282
Extranodal sites, ≥2 vs. < 2	0.623	0.364	1.864 (0.913–3.804)	0.087		0.464	0.313	1.590 (0.861–2.938)	0.139
Ann Arbor Stage, III/IV vs. I/II	0.651	0.334	1.917 (0.996–3.692)	0.052		0.554	0.290	1.740 (0.985–3.074)	0.057
B symptoms, presence vs. absence	0.341	0.335	1.407 (0.730–2.712)	0.308		0.412	0.279	1.510 (0.874–2.610)	0.140
LDH, ≥250 vs. < 250, U/L	0.850	0.353	2.340 (1.171–4.675)	**0.016**		0.664	0.302	1.942 (1.074–3.513)	**0.028**
β2-MG, ≥3 vs. < 3, mg/L	0.970	0.346	2.639 (1.339–5.198)	**0.005**		0.771	0.288	2.161 (1.229–3.802)	**0.007**
PNI, ≤45 vs. > 45	0.822	0.333	2.275 (1.184–4.369)	**0.014**		0.503	0.296	1.653 (0.926–2.954)	0.089
CONUT score, 5–12 vs. 0–4	3.380	0.572	29.385 (9.583–90.102)	**<0.001**		2.527	0.384	12.516 (5.899–26.558)	**<0.001**
IPI				**<0.001**					**<0.001**
0–1	Reference					Reference			
2	1.205	0.427	3.336 (1.445–7.704)	**0.005**		0.820	0.357	2.270 (1.128–4.566)	**0.022**
3	2.217	0.578	9.182 (2.958–28.505)	**<0.001**		1.473	0.522	4.364 (1.570–12.130)	**0.005**
4–5	2.267	0.521	9.651 (3.476–26.794)	**<0.001**		1.630	0.427	5.105 (2.210–11.789)	**<0.001**
KPI				**<0.001**					**<0.001**
0	Reference					Reference			
1	0.156	0.520	1.169 (0.422–3.242)	0.764		0.107	0.386	1.113 (0.522–2.370)	0.782
2	0.553	0.536	1.739 (0.608–4.973)	0.302		0.360	0.421	1.434 (0.628–3.273)	0.393
3–4	2.338	0.559	10.356 (3.459–31.006)	**<0.001**		1.627	0.428	5.090 (2.198–11.786)	**<0.001**
PINK				**<0.001**					**<0.001**
0	Reference					Reference			
1	0.368	0.517	1.445 (0.524–3.980)	0.477		0.003	0.362	1.003 (0.494–2.039)	0.993
2–4	2.661	0.551	14.309 (4.857–42.158)	**<0.001**		1.709	0.362	5.522 (2.719–11.216)	**<0.001**
PINK-E				**<0.001**					**<0.001**
0–1	Reference					Reference			
2	1.382	0.466	3.984 (1.598–9.934)	**0.003**		0.992	0.362	2.695 (1.325–5.484)	**0.006**
3–5	2.709	0.482	15.022 (5.837–38.661)	**<0.001**		2.036	0.346	7.657 (3.884–15.096)	**<0.001**
Therapy									
RT alone	Reference			**0.059**		Reference			**0.402**
CT alone	1.152	0.757	3.163(0.717–13.948)	**0.128**		0.109	0.461	1.115(0.452–2.751)	**0.813**
CRT	0.297	0.769	1.346(0.298–6.077)	**0.699**		0.407	0.304	1.503(0.828–2.727)	**0.180**

ECOG, Eastern Cooperative Oncology Group; BM, bone marrow; LDH, lactate dehydrogenase; β2-MG, beta-2 microglobulin; PNI, prognostic nutritional index; CONUT score, Controlling Nutritional Status score; IPI, international prognostic index; KPI, Korean Prognostic Index; PINK, Prognostic index of natural killer lymphoma; PINK-E, PINK plus Epstein–Barr virus (EBV); OS, overall survival; PFS, progression-free survival; B, coefficient; SE, standard error; HR, Hazard ratio; CI, confidence interval. The bold values mean statistically significant.

### Derivation of a reformative stratification model, CONUT-PINK-E

Since the prognostic capacity of CONUT score has been verified, we attempted to establish a CONUT score-included prognostic model for ENKTL. MVA hinted that PINK-E was another independent predictive marker to OS (PINK-E = 2, HR [95% CI] =3.842 [1.108–13.325], *p* = 0.034; PINK-E = 3/4/5, HR [95% CI] =9.185 [1.888–44.688], *p* = 0.006) and PFS (PINK-E = 2, HR [95% CI] =2.308 [0.964–5.526], *p* = 0.06; PINK-E = 3/4/5, HR [95% CI] =4.535 [1.613–12.751], *p* = 0.004). Meanwhile, PINK-E-estimated survival curves also reverified its qualification as a prognostic marker (Supplementary Figure 1).

Based on the above findings, we reformed PINK-E through incorporating with CONUT score and established an integrated prognostic model, CONUT-PINK-E. The reformative model contains six risk factors, including age ≥ 60 years old, Ann Arbor stage III/IV, distant lymph node involvement, non-nasal type, detectable EBV-DNA in blood and moderate/severe malnutrition (CONUT score ≥ 5). We need to collect 8 variables to get a point of CONUT-PINK-E, including age, Ann Arbor stage, distant lymph node involvement, non-nasal type, detectable EBV-DNA, ALB, TC and ALC. Based on CONUT score, ALB concentrations of ≥3.50 g/dL, 3.00–3.49 g/dL, 2.50–2.99 g/dL, and <2.50 g/dL were scored as 0, 2, 4, and 6 points, TC levels of ≥180 mg/dL, 140–179 mg/dL, 100–139 mg/dL, and <100 mg/dL were endowed with 0, 1, 2, and 3 points, and ALC of ≥1,600/mm^3^, 1,200–1,599/mm^3^, 800–1,199/mm^3^, and <800/mm^3^ were scored as 0, 1, 2, and 3 points, separately. Once the sum of CONUT score arrived 5, it would be regarded as an unfavorable risk factor in the CONUT-PINK-E scoring system. In the novel model, CONUT score ≥ 5 was endowed with two points, PINK-E = 2 with one point and PINK-E = 3/4/5 with two points in accordance with their rounded regression coefficients (B; Figure 1). CONUT-PINK-E was calculated as a sum of points and differentiated patients into five groups: 0 point (no or just one risk factor of PINK-E), 1 point (two risk factors of PINK-E), 2 points (CONUT score ≥ 5 or over 3 risk factors of PINK-E), 3 points (CONUT score ≥ 5 and 2 risk factors of PINK-E), and 4 points (CONUT score ≥ 5 and over 3 risk factors of PINK-E).

### Capacity of CONUT-PINK-E in survival prediction of ENKTL patients

**Figure 2:**
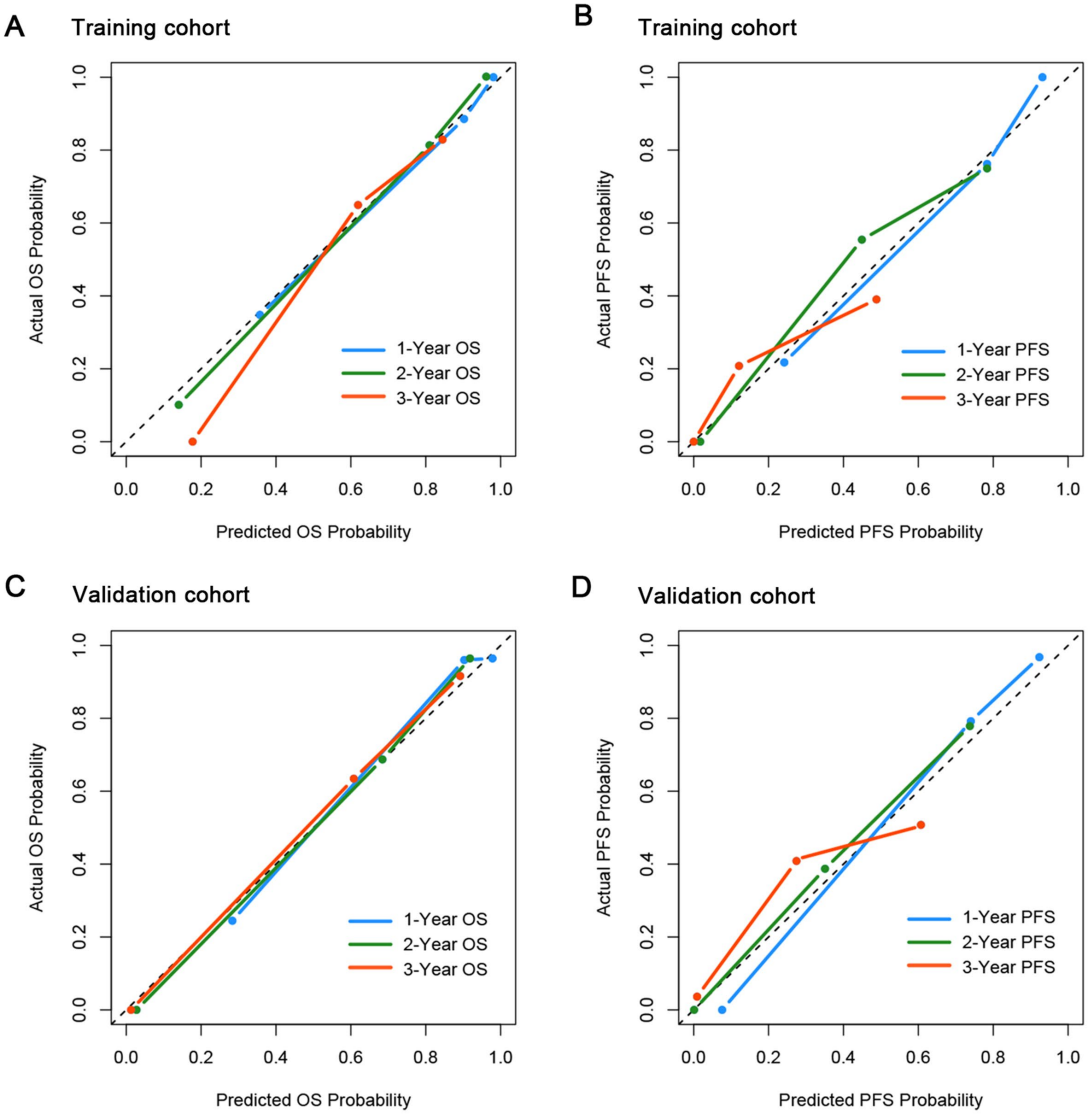
Calibration curves for the probability of OS and PFS in the training and validation cohort. **(A)** for OS in the training cohort; **(B)** for PFS in the training cohort; **(C)** for OS in the validation cohort; **(D)** for PFS in the validation cohort.

To Figure out the predictive ability of CONUT-PINK-E, we assessed its discrimination and calibration in the training cohort and verified them in the external validation cohort. In the training cohort, the Harrell’s C-statistic for OS and PFS prediction was 0.860 (95% CI, 0.821–0.899) and 0.808 (95% CI, 0.760–0.856; Supplementary Table 2), and the calibration plots for the probability of 1-, 2-, and 3-year OS and PFS showed great consistence between the prediction and actual observation (Figures 2A,B). Similarly, in the validation cohort, the Harrell’s C-statistic for OS and PFS prediction was 0.848(95% CI, 0.799–0.897) and 0.811(95% CI, 0.762–0.859), and the calibration plots presented that 1-, 2-, and 3-year prediction perfectly coincided with the actual observation (Figures 2C,D). The results indicated that the predictive capacity of CONUT-PINK-E was encouraging and reliable.

### Prognostic performance of CONUT-PINK-E in survival risk stratification

The overall median OS was 51 (95% CI, 29.3–72.7) months and the 3- and 5-year OS rates were 50.9% and 38.2%, while the median PFS was 23 (95% CI, 17.1–28.9) months and the 3-year PFS rate was 23.2% in the training cohort. The corresponding data of the validation cohort was 48 (95% CI, 34.2–61.8) months, 57.4, 35.3% and 20 (95% CI, 13.0–27.0) months, 30.4%, respectively (Supplementary Table 3). The survival outcomes of two cohorts were close.

**Figure 3:**
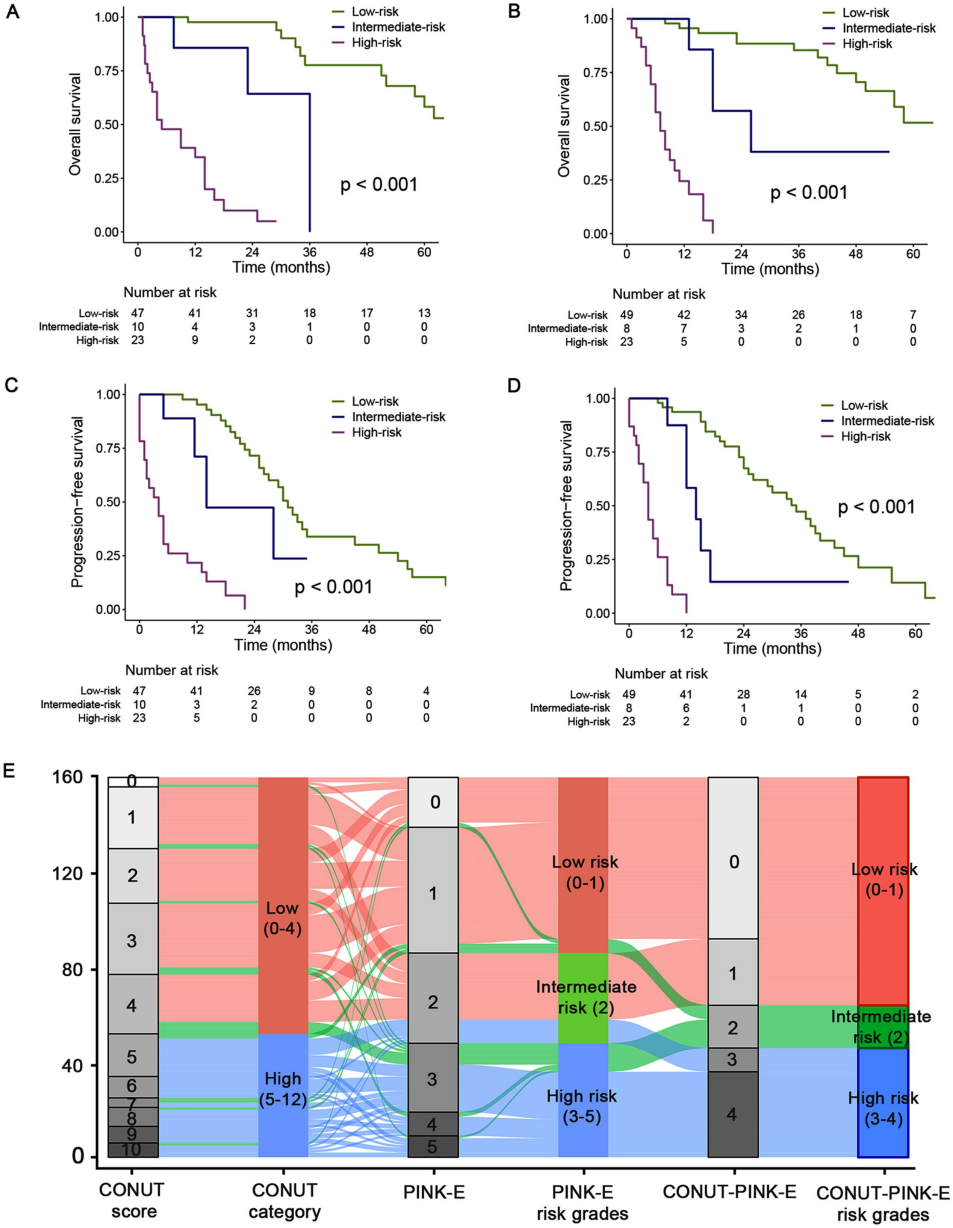
Kaplan–Meier estimated OS and PFS curves of CONUT-PINK-E grades and alluvial plot in the training and validation cohorts. Low risk group with CONUT-PINK-E = 0/1; Intermediate risk group with CONUT-PINK-E = 2; High risk group with CONUT-PINK-E = 3/4. **(A,C)** For OS and PFS in training cohort; **(B,D)** For OS and PFS in the external validation cohort; **(E)** the alluvial plot shows the frequency and relationship between the CONUT-PINK-E risk stratifications and the included risk factors in the total population of training and validation cohort. The width of the ribbons corresponds to the percentage of patients who had the same CONUT score, PINK-E and risk group assigned.

Kaplan–Meier curves were plotted to examine the survival outcomes of patients with different CONUT-PINK-E scores. In the training cohort, the OS of patients with 0, 1, 2, 3, 4 points of CONUT-PINK-E was 74.0 (95%CI, 50.2–97.8), 51.0 (95%CI, 12.8–89.2), 36.0 (95%CI, NA-NA), 14.0 (95%CI, 9.6–18.4), and 4.0 (95%CI, 2.6–5.4), respectively. The corresponding data of PFS was separately 32.0 (95%CI, 26.6–37.4), 26.0 (95%CI, 18.5–33.5), 14.0 (95%CI, 0–29.5), 6.0 (95%CI, 3.9–8.1), and 2.0 (95%CI, 0–5.1). As shown in Supplementary Figure 3A, no significant difference was observed in OS of patients with 0 or 1 point (*p* = 0.118) and patients with 3 or 4 points (*p* = 0.466). While gradual widening of the survival gap between patients with 1 point or 2 points was observed over time, although the sample size of subgroup limited its statistical significance. Patients with 2 or 3 points exhibited significant survival differences (*p* = 0.034). The survival curve of PFS showed a similar trend (Supplementary Figure 3B). Therefore, we divided patients into three risk layers according to survival differences (Figure 3). Patients with 0 or 1 point were included in the low-risk group due to the relatively superior survival, while patients with 2 points were assigned to the intermediate-risk group and patients with 3 or 4 points were classified as high-risk group due to sequentially-shorten survival.

In the training cohort, the median OS of the low-risk group was 74.0 (95%CI, 55.0–93.0) months, significantly superior than 36.0 (NA-NA) and 5.0 (0.3–9.7) months of intermediate- and high-risk group, respectively (*p* < 0.001, Figure 3A; Supplementary Table 4). Afterwards, we further examined the performance of the stratifying rules in the external validation cohort. Significant OS differences (*p* < 0.001) also existed among three risk grades, and the median OS was 72.0 (95%CI, 49.0–95.0), 26.0 (95%CI, 9.6–42.4), and 7.0 (95%CI, 4.7–9.3) months for low-, intermediate- and high-risk, respectively (Figure 3B; Supplementary Table 4). The 3-year OS rate was 77.7 and 85.4% for the low-risk group, while 9.9% and 0 for high-risk group in the training and validation cohort. In addition, the performance of CONUT-PINK-E in PFS risk stratification was equally impressing (Figures 3C,D; Supplementary Table 4). The 2-year PFS rate was 71.5% and 72.6%, 0 and 0 for low- and high-risk group, respectively (Supplementary Table 4). These indicate that CONUT-PINK-E has strong correlation with patients’ survival and can effectively stratify patients.

Alluvial plot exhibited the frequency and relationship between the CONUT-PINK-E and its constituent factors, CONUT and PINK-E, in the total 160 patients. The gray blocks represented the detailed scores and the colorful blocks symbolized the risk groups, while the width of the ribbons corresponded to the percentage of patients who had the same CONUT score, PINK-E and risk group assigned. In the total population, patients in the CONUT-PINK-E low risk group accounted for the majority and no patients with CONUT score < 5 were divided into CONUT-PINK-E high risk group (Figure 3E).

### Performance comparison between CONUT-PINK-E and the current scoring systems

CONUT-PINK-E has been proved capable of serving as an outstanding prognostic model for ENKTL. Naturally, we will compare its performance with the present scoring systems, including IPI, KPI, PINK and PINK-E. Primarily, the discrimination parameter, Harrell’s C-statistic of CONUT-PINK-E was 0.860 (95% CI, 0.821–0.899), significantly higher than 0.744 (95% CI, 0.672–0.816) of IPI (*p* = 0.001), 0.748 (95% CI, 0.681–0.814) of KPI (*p* = 0.001), 0.792 (95% CI, 0.745–0.838) of PINK (*p* = 0.001) and 0.809 (95% CI, 0.762–0.857) of PINK-E (*p* = 0.001) for OS prediction in the training cohort (Supplementary Table 2). The similar superiority in discrimination for OS prediction in the validation cohort and PFS prediction in both training and validation cohort were also confirmed (Supplementary Table 2).

**Figure 4:**
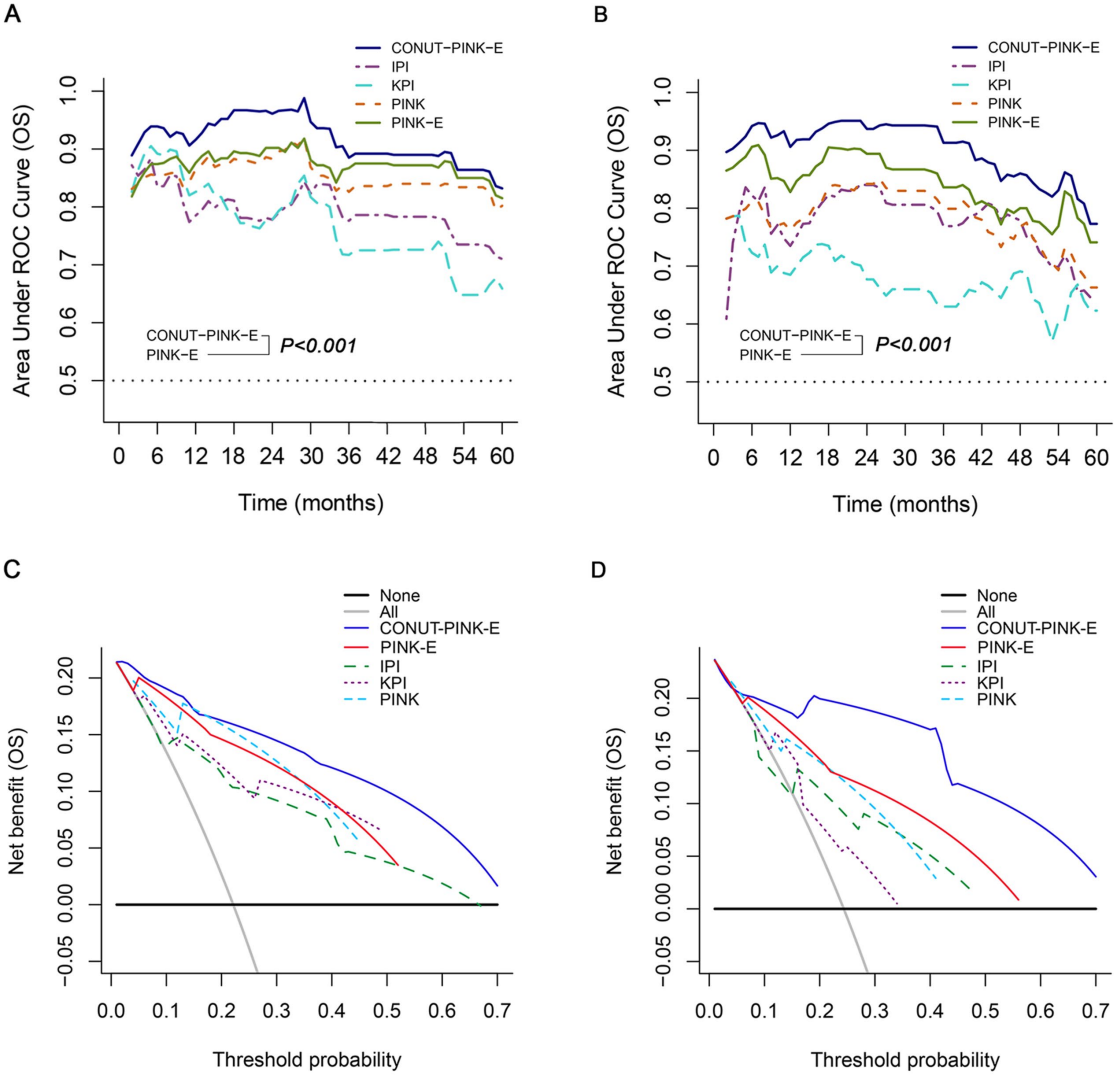
Performance of CONUT-PINK-E in predicting OS in the training and external validation cohorts. Comparison of time-dependent AUCs among CONUT-PINK-E and IPI, KPI, PINK, PINK-E **(A)** in the training cohort, **(B)** in the external validation cohort; Decision curve contrasts of CONUT-PINK-E and IPI, KPI, PINK, PINK-E **(C)** in the training cohort, **(D)** in the external validation cohort.

Time-dependent AUC, a more precise parameter reflecting discrimination, was further measured. The 1- to 5-year time-dependent AUCs with a range for CONUT-PINK-E, IPI, KPI, PINK and PINK-E were ordinally 0.832–0.961, 0.710–0.785 (*p* < 0.001), 0.659–0.826 (*p* < 0.001), 0.802–0.883 (*p* < 0.001) and 0.815–0.902 (*p* < 0.001) in the training cohort (Figure 4A; Supplementary Table 5). The corresponding data in the validation cohort were 0.773–0.937, 0.646–0.840 (*p* < 0.001), 0.623–0.691 (*p* < 0.001), 0.663–0.842 (*p* < 0.001) and 0.741–0.894 (*p* < 0.001, Figure 4B; Supplementary Table 5). The time-dependent AUCs of CONUT-PINK-E in PFS prediction were consistently higher than those of IPI, KPI, PINK and PINK-E in both training and validation cohort (Supplementary Figure 4; Supplementary Table 5). The above results demonstrated that CONUT-PINK-E possessed higher discrimination than the existing models in stratifying ENKTL patients.

The IDI and NRI were calculated to examine the amelioration after incorporating CONUT score with PINK-E and reclassifying the risk grades. CONUT-PINK-E possessed positive IDI and NRI in 1-, 2-, and 3-year OS and PFS prediction in two cohorts, suggesting that CONUT-PINK-E achieved significant improvements compared with the current prognostic tools, which simultaneously indicated that nutritional status played a vital role in the prognosis of ENKTL (Supplementary Tables 6, 7).

DCA was conclusively performed to examine the clinical utility of CONUT-PINK-E, since decision curve could graphically present the clinical value of a prognostic tool based on a continuum of potential thresholds for risk of death (the x-axis) and the net benefit of using the model to stratify patients relative to the assumption that no patient would be dead (the y-axis). The result demonstrated CONUT-PINK-E brought higher net benefits than IPI, KPI, PINK and PINK-E in forecasting OS and PFS of ENKTL patients (OS: Figures 4C,D; PFS: Supplementary Figure 4).

## Discussion

In this study, we provided new arguments to support the correlation between malnutrition and lymphoma, where malnutrition was strongly associated with inferior survival outcomes of patients with ENKTL. CONUT score, as an index reflecting long-term nutritional status, was a powerful tool to screen prognosis-related malnutrition in ENKTL, outperforming the PNI score. Additionally, CONUT score ≥ 5 served as an independent marker indicating unfavorable outcomes, which further confirmed the critical role of malnutrition in ENKTL and hinted us that combining CONUT score with another independent factor, PINK-E, might get better stratifying performance. Thus, the first nutrition evaluation-incorporated risk stratifying system for ENKTL patients, CONUT-PINK-E, was delivered. CONUT-PINK-E was composed of simple laboratorial and imaging parameters, reliable and accessible, might provide helpful reference for individualized nutritional and metabolic care.

Nutrition has been gradually regarded as a critical factor involved in the progression of malignancies (20), since nutrition could change drug metabolic pathways and interfere the expression of drug transporters, resulting in slower clearance of anticancer drugs and enhancive treatment-related toxicities (21). Nutrition status can be assessed by various indicators, including but not limited to body weight, triceps skin fold thickness, mid-arm muscle circumference, BMI, ALB, ALC, transferrin and creatinine to height ratio (15, 22). Nonetheless, previous studies verified that parameters, like BMI, ALC and ALB, were not significant enough to serve as independent survival indicators against the CONUT score in various cancers (14, 23, 24). PNI, a serology-derived nutrition parameter, was originally developed for perioperative patients and afterwards generalized into patients with malignancies, which was also found less valuable than CONUT score in survival prediction for multiple malignancies (25, 26). Consistent with fore-mentioned reports, our study also found that CONUT score was a more comprehensive and efficient tool to detect malnutrition for survival prediction in ENKTL, of which ALB mainly reflects the patient’s nutritional status, ALC serves as a marker of nutrition and immune response and TC has been verified significantly associated with the long-term prognosis of mature T- and NK-cell neoplasms in our previous report (27).

Previously, numerous prognostic tools have been proposed for ENKTL, such as IPI (28), KPI (29) developed in CHOP or CHOP-like era (30) and PINK or PINK-E (31) derived in non-anthracycline-based chemotherapy era. However, IPI and KPI became less useful with the abandonment of anthracycline-containing regimen (32). PINK and PINK-E behaved well but presented limitations in guiding the suitable therapeutic regimens (33). Our study also showed that PINK-E performed best among the existing prognostic systems. Nevertheless, the performance of PINK-E acquired further amelioration by incorporating with CONUT score. CONUT-PINK-E behaved better in discrimination, calibration and clinical net benefit than IPI, KPI, PINK and PINK-E. The high-risk patients identified by CONUT-PINK-E presented worse survival than those identified by the existed models, hinting us their treatment schedules should be more deliberative. The treatment landscape for ENKTL has evolved in last decades, with accumulating evidence supporting systemic L-Asparaginase incorporated non-anthracycline-based chemotherapy was an optimal choice for advanced-stage and relapsed or refractory ENKTL (2, 31). Despite this, a portion of advanced patients face the dilemma aborting the treatment schedules due to the intolerable toxicities (34). The CONUT-PINK-E score targeted this challenge and provided vital theoretical guidance for clinical decision-making. Although few nutrition interventional studies for specific types of hematological tumors were being conducted (7), suggestions from clinical nutritionists and multidisciplinary consultations can favor to develop more rational treatment schedules, helping patients to endure long-term intensive chemotherapy. Furthermore, with the awareness of the relationship between malnutrition and malignancies, increasing number of nutrition interventional studies would be conducted and nutrition intervention schemes would be listed in the guidelines of cancer treatment.

Nevertheless, the limitations should be addressed. First, CONUT-PINK-E was derived from retrospective study, which had its inherent biases. Second, the sample size was relatively modest, although independent external validation was performed to increase the credibility. Third, fewer patients in this study experienced immunotherapy or HSCT, making it impossible to compare the prognostic influence of different therapies for patients under the same risk stratification.

In future, with the increasement of sample size and therapy options, subgroup analysis of different therapies for patients under the same risk stratification might be conducted, which could help to identify the specific subgroups who would benefit most from CONUT-PINK-E system.

In conclusion, it is the first time that CONUT score was recognized to act as an independent prognostic parameter in newly diagnosed ENKTL patients. More importantly, the first risk stratification system covering nutritional status assessment for ENKTL patients, CONUT-PINK-E, was derived from the data of two centers in China. The data in our study, including treatment therapies and survival outcomes, were consistent with the precedent large-scale, real-world reports. Based on that, CONUT-PINK-E presented better discrimination, calibration, prognostic performance, stability than the existed prognostic models, suggesting that it would provide convinced guidance for ENKTL patients’ clinical decision-making.

## Data availability statement

The original contributions presented in the study are included in the article/Supplementary material, further inquiries can be directed to the corresponding authors on a reasonable request.

## Ethics statement

The studies involving human participants were reviewed and approved by the Medical Ethical Committee of Shandong Provincial Hospital Affiliated to Shandong University and the Medical Ethical Committee of Affiliated Hospital of Qingdao University. Written informed consent for participation was not required for this study in accordance with the national legislation and the institutional requirements.

## Author contributions

TL: study concept and design, data analysis, and writing the manuscript. XS and YL: data collection of the external validation cohort. XG, MD, and XF: data acquisition of the training cohort. YC, XC, and SH: patients’ follow-up. FL: study concept and design. XW and XZ: study supervision and manuscript revision. All authors contributed to the article and approved the submitted version.

## Funding

This study was supported by National Natural Science Foundation (nos. 82170189, 82070203, 81800194, and 81770210), Key Research and Development Program of Shandong Province (no. 2018CXGC1213), Development Project of Youth Innovation Teams in Colleges and Universities of Shandong Province (no. 2020KJL006), China Postdoctoral Science Foundation (nos. 2021T140422 and 2020M672103), Translational Research Grant of NCRCH (nos. 2021WWB02 and 2020ZKMB01), Shandong Provincial Natural Science Foundation (nos. ZR2021YQ51 and ZR2020MH124), Technology Development Project of Jinan City (no. 202134034), Taishan Scholars Program of Shandong Province, Shandong Provincial Engineering Research Center of Lymphoma, and Academic Promotion Programme of Shandong First Medical University (nos. 2019QL018 and 2020RC006).

## Conflict of interest

The authors declare that the research was conducted in the absence of any commercial or financial relationships that could be construed as a potential conflict of interest.

## Publisher’s note

All claims expressed in this article are solely those of the authors and do not necessarily represent those of their affiliated organizations, or those of the publisher, the editors and the reviewers. Any product that may be evaluated in this article, or claim that may be made by its manufacturer, is not guaranteed or endorsed by the publisher.
