# Supplementary material for: Derivation and validation of a nutrition-covered prognostic scoring system for extranodal NK/T-cell lymphoma
Source: Front Nutr. 2023 May 12;10:1080181. doi: 10.3389/fnut.2023.1080181 (PMC10213411; doi:10.3389/fnut.2023.1080181)
Supplement: Supplementary file 1 [file Data_Sheet_1.docx]

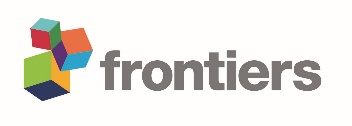
Supplementary Material

**Supplementary Table 1. Controlling Nutritional Status (CONUT) score criteria.**

| Variables | **Nutritional status(score)** | | | |
| --- | --- | --- | --- | --- |
|  | Normal  (0-1) | Mild  malnutrition  (2-4) | Moderate  Malnutrition  (5-8) | Severe  malnutrition  (9-12) |
| ALB (g/dL) | 3.50-4.50(0) | 3.00-3.49(2) | 2.50-2.90(4) | ＜2.50(6) |
| TC (mg/dL) | ＞180(0) | 140-180(1) | 100-139(2) | ＜100(3) |
| ALC (/ml) | ＞1600(0) | 1200-1599(1) | 800-1199(2) | ＜800(3) |

**Abbreviations:** ALB: serum albumin; TC: serum total cholesterol; ALC: absolute lymphocyte count.

**Supplementary Figure 1. Kaplan-Meier estimated OS and PFS curves of CONUT score and PINK-E in the training cohort. (A: CONUT OS; B: CONUT PFS; C: PINK-E OS; D: PINK-E PFS)**


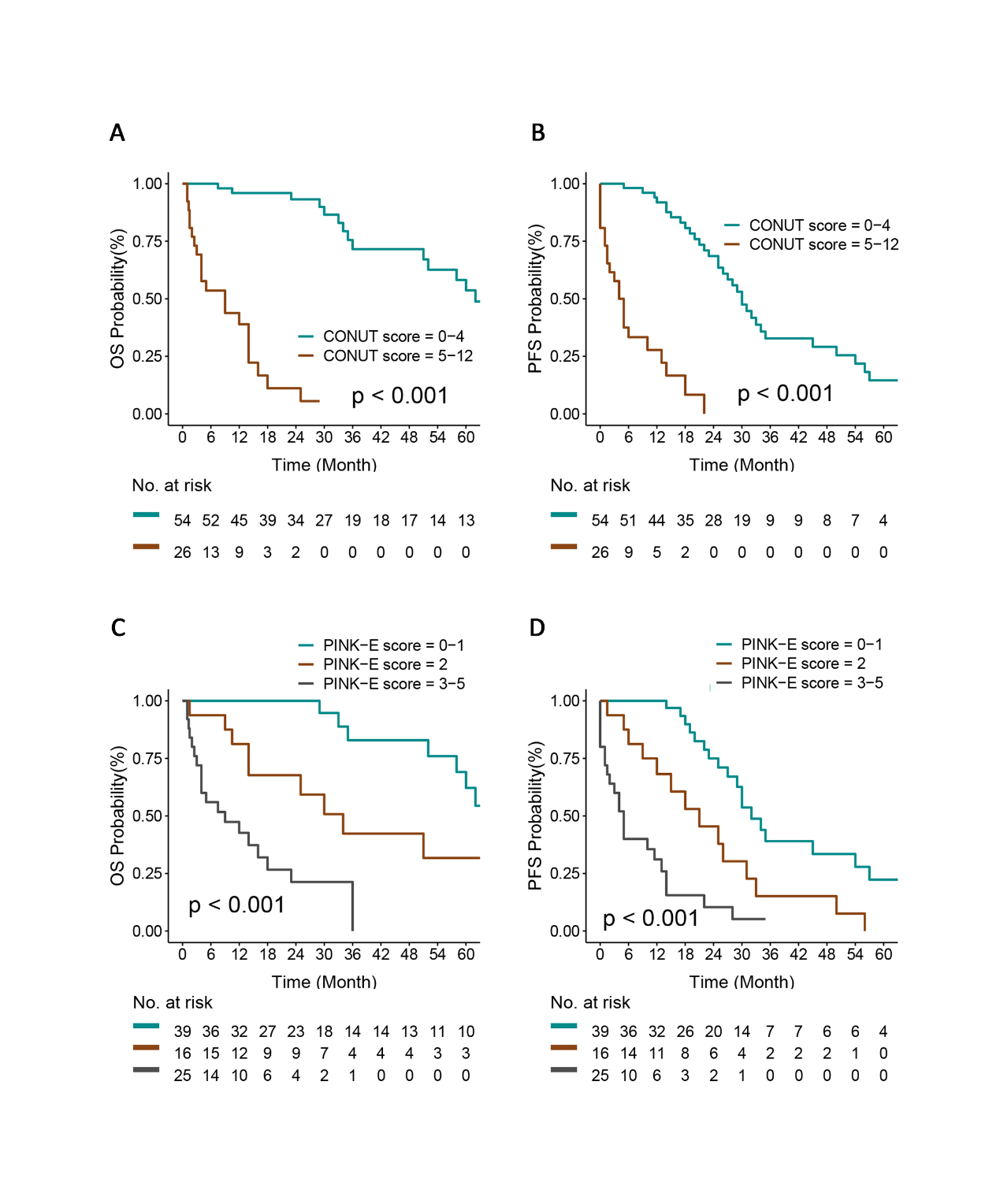


**Supplementary Table 2. Discrimination of CONUT-PINK-E and IPI, KPI, PINK, PINK-E in predicting OS/PFS.**

|  | **Cohort** | **Scoring systems** | **Harrell’s c-index** | **Low** | **Up** | **P (compared)** |
| --- | --- | --- | --- | --- | --- | --- |
| **OS** | **Training Cohort** | CONUT-PINK-E | 0.860 | 0.821 | 0.899 | *Reference* |
|  |  | IPI | 0.744 | 0.672 | 0.816 | 0.001 |
|  |  | KPI | 0.748 | 0.681 | 0.814 | 0.001 |
|  |  | PINK | 0.792 | 0.745 | 0.838 | 0.001 |
|  |  | PINK-E | 0.809 | 0.762 | 0.857 | 0.001 |
|  | **Validation Cohort** | CONUT-PINK-E | 0.848 | 0.799 | 0.897 | *Reference* |
|  |  | IPI | 0.716 | 0.640 | 0.791 | 0.001 |
|  |  | KPI | 0.661 | 0.579 | 0.743 | <0.001 |
|  |  | PINK | 0.747 | 0.688 | 0.806 | <0.001 |
|  |  | PINK-E | 0.793 | 0.738 | 0.847 | 0.002 |
| **PFS** | **Training Cohort** | CONUT-PINK-E | 0.808 | 0.760 | 0.856 | *Reference* |
|  |  | IPI | 0.684 | 0.618 | 0.749 | 0.001 |
|  |  | KPI | 0.695 | 0.626 | 0.763 | 0.001 |
|  |  | PINK | 0.725 | 0.672 | 0.778 | <0.001 |
|  |  | PINK-E | 0.772 | 0.723 | 0.821 | 0.010 |
|  | **Validation Cohort** | CONUT-PINK-E | 0.811 | 0.762 | 0.859 | *Reference* |
|  |  | IPI | 0.697 | 0.635 | 0.760 | 0.002 |
|  |  | KPI | 0.653 | 0.583 | 0.723 | <0.001 |
|  |  | PINK | 0.711 | 0.659 | 0.764 | <0.001 |
|  |  | PINK-E | 0.755 | 0.704 | 0.805 | 0.001 |

**Abbreviations:** OS: overall survival; PFS: progression-free survival; CONUT: Controlling Nutritional Status score; CONUT-PINK-E: CONUT-modified PINK-E; IPI: international prognostic index, KPI: Korean Prognostic Index; PINK: Prognostic index of natural killer lymphoma; PINK-E: PINK plus Epstein-Barr virus (EBV)

**Supplementary Table 3. OS/PFS probability and median OS/PFS time of the training and validation cohort.**

|  | **Training Cohort (n=80)** | | **Validation Cohort (n=80)** | | **p value** |
| --- | --- | --- | --- | --- | --- |
|  | **Year rate and median survival time** | | **Year rate and median survival time** | |  |
| **OS** | 1-year Probability | 77.9% | 1-year Probability | 75.6% | - |
|  | 3-year Probability | 50.9% | 3-year Probability | 57.4% | - |
|  | 5-year Probability | 38.2% | 5-year Probability | 35.3% | - |
|  | Median survival time (months) | 51.0 (29.3-72.7) | Median survival time (months) | 48.0 (34.2-61.8) | 0.804 |
| **PFS** | 1-year Probability | 71.5% | 1-year Probability | 62.8% | - |
|  | 2-year Probability | 48.6% | 2-year Probability | 42.6% | - |
|  | 3-year Probability | 23.2% | 3-year Probability | 30.4% | - |
|  | Median survival time (months) | 23.0 (17.1-28.9） | Median survival time (months) | 20.0 (13.0-27.0) | 0.806 |

**Abbreviations:** OS: overall survival; PFS: progression-free survival; NA: Not available.

**Supplementary Figure 2. Kaplan-Meier estimated OS (A) and PFS (B) curves in the training and validation cohort.**
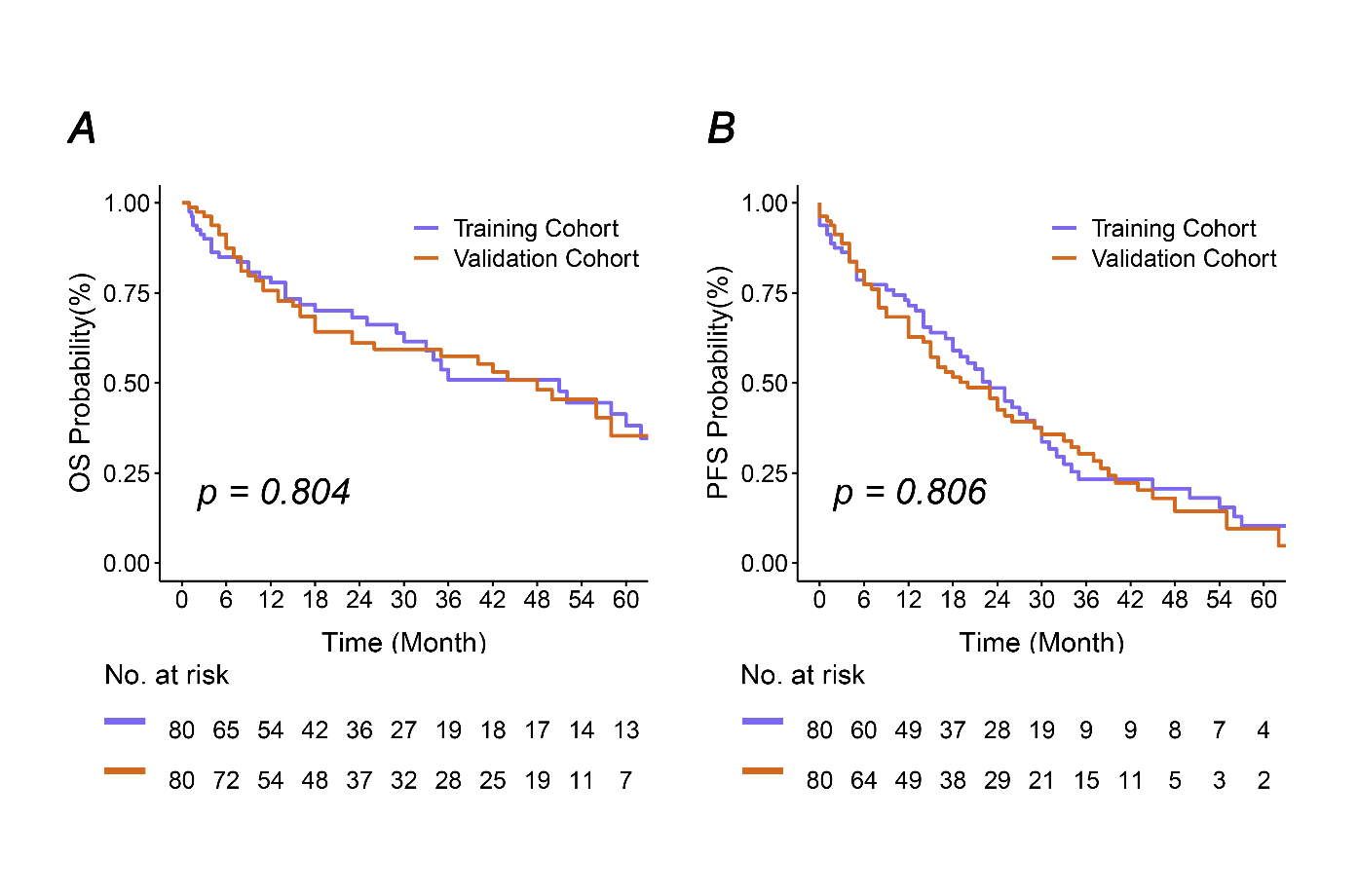


**Supplementary Figure 3. Kaplan-Meier estimated OS and PFS curves of CONUT-PINK-E scores in the training cohort.**
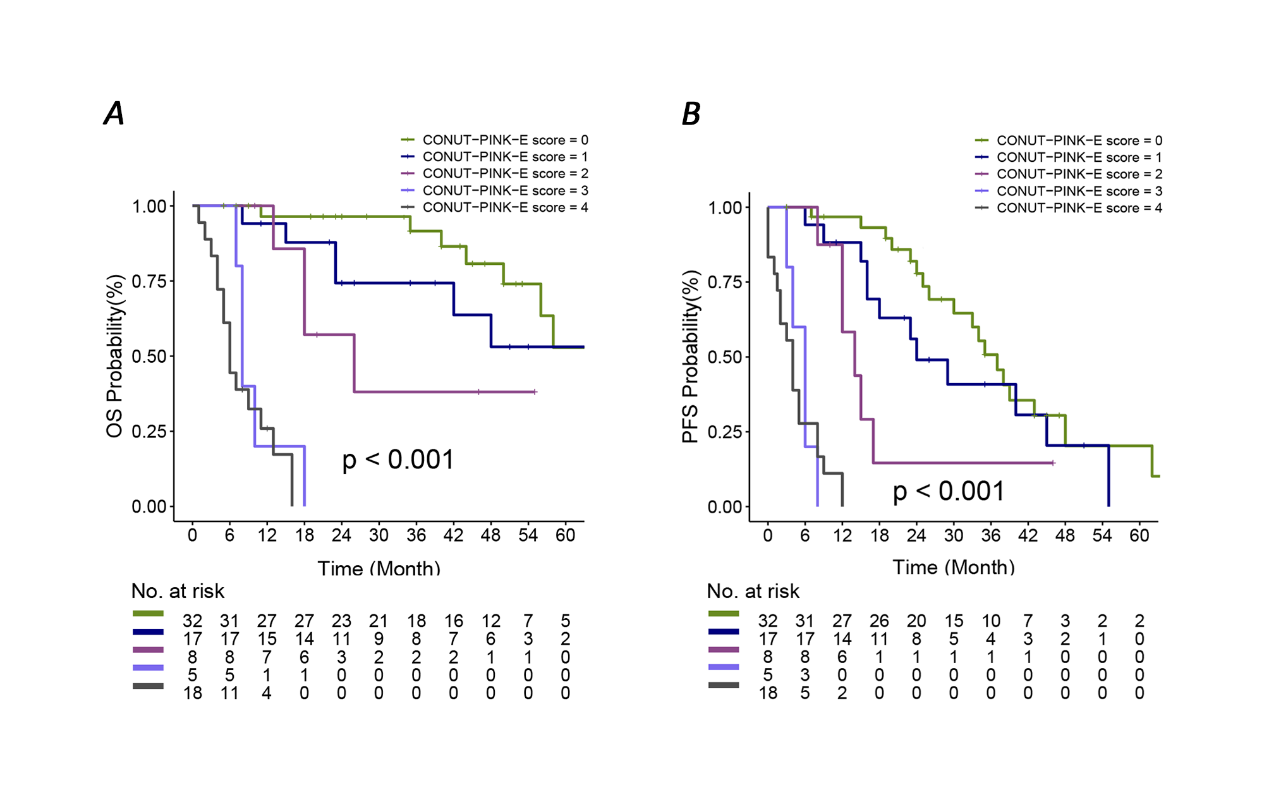


**Supplementary Table 4.** **OS/PFS probability and median OS/PFS time of CONUT-PINK-E in the training and validation cohort.**

|  | **Risk Grades** | **Training Cohort (n=80)** | | **Validation Cohort (n=80)** | |
| --- | --- | --- | --- | --- | --- |
|  |  | **Year rate and Median survival time** | | **Year rate and Median survival time** | |
| **OS** | **Low (0-1)**  **(n=47/49)** | 1-year | 97.7% | 1-year | 95.6% |
|  |  | 3-year | 77.7% | 3-year | 85.4% |
|  |  | 5-year | 58.2% | 5-year | 51.6% |
|  |  | Median OS (months) | 74.0 (55.0-93.0) | Median OS (months) | 72.0 (49.0-95.0) |
|  | **Intermediate (2)**  **(n=10/8)** | 1-year | 85.7% | 1-year | 100.0% |
|  |  | 3-year | 0.0% | 3-year | 38.1% |
|  |  | 5-year | 0.0% | 5-year | NA |
|  |  | Median OS (months) | 36.0 (NA-NA) | Median OS (months) | 26.0 (9.6-42.4) |
|  | **High (3-4) (n=23/23)** | 1-year | 34.8% | 1-year | 24.5% |
|  |  | 3-year | 9.9% | 3-year | 0.0% |
|  |  | 5-year | NA | 5-year | 0.0% |
|  |  | Median OS (months) | 5.0 (0.3-9.7) | Median OS (months) | 7.0 (4.7-9.3) |
|  | **P value** |  | <0.001 |  | <0.001 |
| **PFS** | **Low (0-1)**  **(n=47/49)** | 1-year | 90.5% | 1-year | 93.7% |
|  |  | 2-year | 71.5% | 2-year | 72.6% |
|  |  | 3-year | 33.9% | 3-year | 47.3% |
|  |  | MedianPFS (months) | 31.0 (27.1-34.9) | Median PFS (months) | 35.0 (25.9-44.1) |
|  | **Intermediate (2)**  **(n=10/8)** | 1-year | 71.1% | 1-year | 58.3% |
|  |  | 2-year | 47.4% | 2-year | 14.6% |
|  |  | 3-year | NA | 3-year | 14.6% |
|  |  | Median PFS (months) | 14.0 (0.0-29.5) | Median PFS (months) | 14.0 (8.9-19.1) |
|  | **High (3-4) (n=23/23)** | 1-year | 21.7% | 1-year | 0.0% |
|  |  | 2-year | 0.0% | 2-year | 0.0% |
|  |  | 3-year | 0.0% | 3-year | 0.0% |
|  |  | Median PFS (months) | 4.0 (0.9-7.1) | Median PFS (months) | 4.0 (2.8-5.2) |
|  | **P value** |  | <0.001 |  | <0.001 |

**Abbreviations:** OS: overall survival; PFS: progression-free survival; CONUT: Controlling Nutritional Status score; PINK-E: PINK plus Epstein-Barr virus (EBV); CONUT-PINK-E: CONUT-modified PINK-E.

**Supplementary Figure 4. Time-dependent AUCs and Decision curves of PFS in the training and validation cohort.**
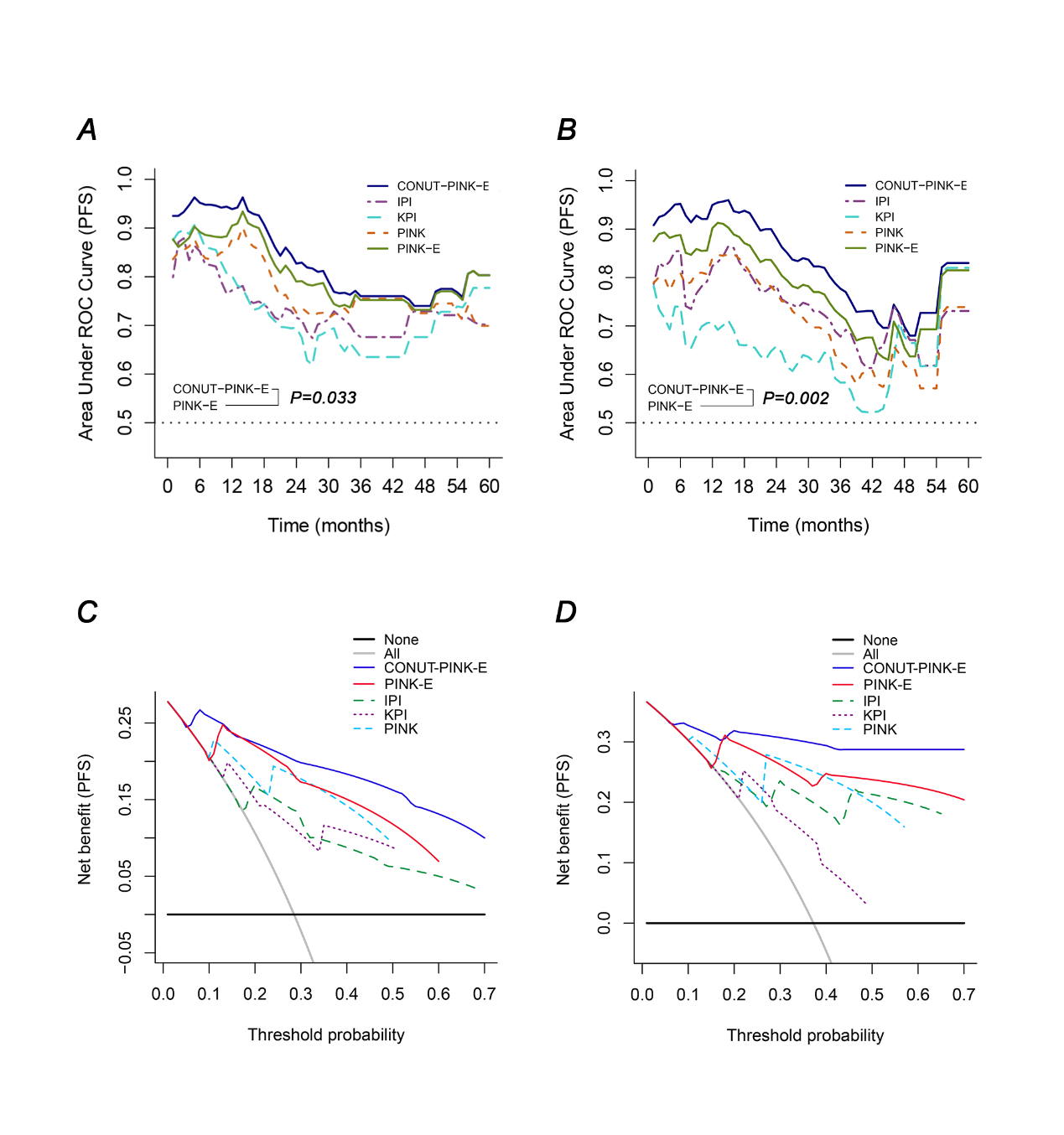


**Supplementary Table 5. Time-dependent AUCs of CONUT-PINK-E and IPI, KPI, PINK, PINK-E for OS/PFS predicting in the training and validation cohort.**

|  | **Cohort** | **Year rate** | **CONUT-PINK-E** | **IPI** | **KPI** | **PINK** | **PINK-E** |
| --- | --- | --- | --- | --- | --- | --- | --- |
| **OS** | **Training Cohort (n=80)** | 1-year | 0.916 | 0.785 | 0.826 | 0.865 | 0.876 |
|  |  | 2-year | 0.961 | 0.778 | 0.778 | 0.883 | 0.902 |
|  |  | 3-year | 0.885 | 0.776 | 0.717 | 0.826 | 0.867 |
|  |  | 4-year | 0.890 | 0.783 | 0.726 | 0.840 | 0.872 |
|  |  | 5-year | 0.832 | 0.710 | 0.659 | 0.802 | 0.815 |
|  | **P-value** | | *Reference* | <0.001 | <0.001 | <0.001 | <0.001 |
|  | **Validation Cohort (n=80)** | 1-year | 0.906 | 0.735 | 0.685 | 0.762 | 0.828 |
|  |  | 2-year | 0.937 | 0.840 | 0.677 | 0.842 | 0.894 |
|  |  | 3-year | 0.913 | 0.769 | 0.630 | 0.799 | 0.836 |
|  |  | 4-year | 0.860 | 0.779 | 0.691 | 0.768 | 0.800 |
|  |  | 5-year | 0.773 | 0.646 | 0.623 | 0.663 | 0.741 |
|  | **P-value** | | *Reference* | <0.001 | <0.001 | <0.001 | <0.001 |
| **PFS** | **Training Cohort (n=80)** | 1-year | 0.939 | 0.772 | 0.802 | 0.877 | 0.905 |
|  |  | 2-year | 0.827 | 0.716 | 0.695 | 0.726 | 0.790 |
|  |  | 3-year | 0.760 | 0.676 | 0.635 | 0.755 | 0.752 |
|  |  | 4-year | 0.740 | 0.729 | 0.676 | 0.725 | 0.732 |
|  |  | 5-year | 0.803 | 0.702 | 0.777 | 0.699 | 0.803 |
|  | **P-value** | | *Reference* | <0.001 | <0.001 | <0.001 | 0.033 |
|  | **Validation Cohort (n=80)** | 1-year | 0.950 | 0.824 | 0.708 | 0.840 | 0.902 |
|  |  | 2-year | 0.882 | 0.783 | 0.658 | 0.769 | 0.821 |
|  |  | 3-year | 0.777 | 0.678 | 0.583 | 0.625 | 0.722 |
|  |  | 4-year | 0.699 | 0.690 | 0.678 | 0.620 | 0.655 |
|  |  | 5-year | 0.830 | 0.731 | 0.820 | 0.739 | 0.815 |
|  | **P-value** | | *Reference* | <0.001 | <0.001 | <0.001 | 0.002 |

**Abbreviations:** AUCs: Areas under the ROC curve (ROC: Receiver operating characteristic curve); OS: overall survival; PFS: progression-free survival; CONUT: Controlling Nutritional Status score; CONUT-PINK-E: CONUT-modified PINK-E; IPI: international prognostic index, KPI: Korean Prognostic Index; PINK: Prognostic index of natural killer lymphoma; PINK-E: PINK plus Epstein-Barr virus (EBV).

**Supplementary Table 6. IDI and NRI of CONUT-PINK-E versus PINK-E, IPI, KPI, PINK for OS prediction in the training and validation cohort.**

| **Cohort** | **Year rate** | **Staging Criteria** | **IDI (95%CI)** | **P-value** | **NRI (95%CI)** | **P-value** |
| --- | --- | --- | --- | --- | --- | --- |
| **Training Cohort (n=80)** | 1-year | CONUT-PINK-E |  |  |  |  |
|  |  | CONUT-PINK-E—PINK-E | 0.167 (0.019-0.315) | 0.020 | 0.699 (0.407-0.856) | 0.014 |
|  |  | CONUT-PINK-E—IPI | 0.263 (0.037-0.464) | 0.020 | 0.639 (0.249-0.808) | 0.002 |
|  |  | CONUT-PINK-E—KPI | 0.268 (0.079-0.432) | 0.004 | 0.658 (0.323-0.824) | 0.004 |
|  |  | CONUT-PINK-E—PINK | 0.202 (0.016-0.358) | 0.038 | 0.676 (0.077-0.842) | 0.028 |
|  | 2-year | CONUT-PINK-E |  |  |  |  |
|  |  | CONUT-PINK-E—PINK-E | 0.233 (0.047-0.402) | 0.010 | 0.799 (0.527-0.931) | 0.020 |
|  |  | CONUT-PINK-E—IPI | 0.414 (0.217-0.583) | <0.001 | 0.732 (0.444-0.894) | <0.001 |
|  |  | CONUT-PINK-E—KPI | 0.433 (0.210-0.587) | <0.001 | 0.677 (0.405-0.913) | <0.001 |
|  |  | CONUT-PINK-E—PINK | 0.278 (0.056-0.452) | 0.010 | 0.740 (0.165-0.909) | 0.012 |
|  | 3-year | CONUT-PINK-E |  |  |  |  |
|  |  | CONUT-PINK-E—PINK-E | 0.119 (0.005-0.266) | 0.060 | 0.629 (0.199-0.808) | 0.332 |
|  |  | CONUT-PINK-E—IPI | 0.282 (0.110-0.466) | 0.002 | 0.466 (0.189-0.778) | 0.008 |
|  |  | CONUT-PINK-E—KPI | 0.317 (0.137-0.474) | 0.004 | 0.557 (0.156-0.775) | 0.002 |
|  |  | CONUT-PINK-E—PINK | 0.143 (0.004-0.267) | 0.046 | 0.371 (0.034-0.703) | 0.024 |
| **Validation Cohort (n=80)** | 1-year | CONUT-PINK-E |  |  |  |  |
|  |  | CONUT-PINK-E—PINK-E | 0.267 (0.121-0.390) | <0.001 | 0.761 (0.556-0.892) | <0.001 |
|  |  | CONUT-PINK-E—IPI | 0.374 (0.205-0.515) | <0.001 | 0.655 (0.439-0.862) | <0.001 |
|  |  | CONUT-PINK-E—KPI | 0.458 (0.237-0.630) | <0.001 | 0.761 (0.495-0.920) | <0.001 |
|  |  | CONUT-PINK-E—PINK | 0.367 (0.173-0.521) | <0.001 | 0.761 (0.394-0.901) | 0.002 |
|  | 2-year | CONUT-PINK-E |  |  |  |  |
|  |  | CONUT-PINK-E—PINK-E | 0.200 (0.058-0.357) | 0.004 | 0.676 (0.462-0.842) | 0.024 |
|  |  | CONUT-PINK-E—IPI | 0.330 (0.147-0.507) | <0.001 | 0.529 (0.285-0.795) | <0.001 |
|  |  | CONUT-PINK-E—KPI | 0.520 (0.334-0.668) | <0.001 | 0.613 (0.429-0.861) | <0.001 |
|  |  | CONUT-PINK-E—PINK | 0.319 (0.122-0.520) | 0.002 | 0.541 (0.205-0.817) | 0.002 |
|  | 3-year | CONUT-PINK-E |  |  |  |  |
|  |  | CONUT-PINK-E—PINK-E | 0.201 (0.073-0.358) | <0.001 | 0.682 (0.055-0.841) | 0.030 |
|  |  | CONUT-PINK-E—IPI | 0.337 (0.168-0.500) | <0.001 | 0.567 (0.334-0.787) | <0.001 |
|  |  | CONUT-PINK-E—KPI | 0.504 (0.330-0.654) | <0.001 | 0.674 (0.515-0.850) | <0.001 |
|  |  | CONUT-PINK-E—PINK | 0.305 (0.111-0.488) | 0.008 | 0.432 (0.200-0.825) | 0.002 |

**Abbreviations:** IDI: integrated discrimination improvement; NRI: net reclassification index; OS: overall survival; CONUT: Controlling Nutritional Status score; CONUT-PINK-E: CONUT-modified PINK-E; IPI: international prognostic index, KPI: Korean Prognostic Index; PINK: Prognostic index of natural killer lymphoma; PINK-E: PINK plus Epstein-Barr virus (EBV).

**Supplementary Table 7. IDI and NRI of CONUT-PINK-E versus PINK-E, IPI, KPI, PINK for PFS prediction in the training and validation cohort.**

| **Cohort** | **Year rate** | **Staging Criteria** | **IDI (95%CI)** | **P-value** | **NRI (95%CI)** | **P-value** |
| --- | --- | --- | --- | --- | --- | --- |
| **Training Cohort (n=80)** | 1-year | CONUT-PINK-E |  |  |  |  |
|  |  | CONUT-PINK-E—PINK-E | 0.197 (0.021-0.362) | 0.020 | 0.743 (0.104-0.889) | 0.032 |
|  |  | CONUT-PINK-E—IPI | 0.343 (0.107-0.547) | 0.002 | 0.677 (0.331-0.856) | <0.001 |
|  |  | CONUT-PINK-E—KPI | 0.374 (0.187-0.549) | <0.001 | 0.662 (0.448-0.874) | <0.001 |
|  |  | CONUT-PINK-E—PINK | 0.292 (0.105-0.443) | 0.004 | 0.743 (0.219-0.898) | 0.006 |
|  | 2-year | CONUT-PINK-E |  |  |  |  |
|  |  | CONUT-PINK-E—PINK-E | 0.113 (0.011-0.240) | 0.038 | 0.569 (0.033-0.726) | 0.036 |
|  |  | CONUT-PINK-E—IPI | 0.256 (0.097-0.437) | 0.002 | 0.375 (0.123-0.697) | 0.016 |
|  |  | CONUT-PINK-E—KPI | 0.288 (0.119-0.446) | 0.002 | 0.450 (0.155-0.676) | 0.008 |
|  |  | CONUT-PINK-E—PINK | 0.221 (0.107-0.333) | 0.002 | 0.569 (0.207-0.714) | 0.010 |
|  | 3-year | CONUT-PINK-E |  |  |  |  |
|  |  | CONUT-PINK-E—PINK-E | 0.008 (-0.043-0.096) | 0.805 | 0.039 (-0.272-0.504) | 1.000 |
|  |  | CONUT-PINK-E—IPI | 0.117 (0.008-0.233) | 0.032 | 0.361 (0.002-0.609) | 0.046 |
|  |  | CONUT-PINK-E—KPI | 0.136 (0.013-0.250) | 0.026 | 0.433 (0.048-0.699) | 0.026 |
|  |  | CONUT-PINK-E—PINK | 0.017 (-0.101-0.135) | 0.771 | 0.009 (-0.246-0.505) | 1.000 |
| **Validation Cohort (n=80)** | 1-year | CONUT-PINK-E |  |  |  |  |
|  |  | CONUT-PINK-E—PINK-E | 0.299 (0.158-0.445) | <0.001 | 0.756 (0.560-0.895) | <0.001 |
|  |  | CONUT-PINK-E—IPI | 0.399 (0.225-0.549) | <0.001 | 0.673 (0.438-0.870) | <0.001 |
|  |  | CONUT-PINK-E—KPI | 0.520 (0.335-0.668) | <0.001 | 0.715 (0.456-0.876) | <0.001 |
|  |  | CONUT-PINK-E—PINK | 0.409 (0.219-0.578) | <0.001 | 0.756 (0.377-0.885) | <0.001 |
|  | 2-year | CONUT-PINK-E |  |  |  |  |
|  |  | CONUT-PINK-E—PINK-E | 0.131 (0.041-0.282) | 0.002 | 0.591 (0.411-0.735) | 0.024 |
|  |  | CONUT-PINK-E—IPI | 0.207 (0.070-0.355) | 0.002 | 0.509 (0.187-0.733) | 0.004 |
|  |  | CONUT-PINK-E—KPI | 0.333 (0.178-0.472) | <0.001 | 0.627 (0.355-0.788) | <0.001 |
|  |  | CONUT-PINK-E—PINK | 0.229 (0.102-0.370) | <0.001 | 0.495 (0.270-0.720) | <0.001 |
|  | 3-year | CONUT-PINK-E |  |  |  |  |
|  |  | CONUT-PINK-E—PINK-E | 0.066 (0.008-0.163) | 0.020 | 0.460 (0.115-0.602) | 0.112 |
|  |  | CONUT-PINK-E—IPI | 0.137 (0.003-0.262) | 0.056 | 0.438 (0.009-0.641) | 0.044 |
|  |  | CONUT-PINK-E—KPI | 0.197 (0.054-0.339) | 0.008 | 0.395 (0.107-0.642) | 0.008 |
|  |  | CONUT-PINK-E—PINK | 0.180 (0.080-0.273) | <0.001 | 0.567 (0.298-0.757) | 0.004 |

**Abbreviations:** IDI: integrated discrimination improvement; NRI: net reclassification index; PFS: progression-free survival; CONUT: Controlling Nutritional Status score; CONUT-PINK-E: CONUT-modified PINK-E; IPI: international prognostic index, KPI: Korean Prognostic Index; PINK: Prognostic index of natural killer lymphoma; PINK-E: PINK plus Epstein-Barr virus (EBV).
